# Chemistry, as Exemplifying the Wisdom and Beneficence of God

**Published:** 1846-10

**Authors:** 


### REVIEWS.

---

ART. V.—*Chemistry, as Exemplifying the Wisdom and Beneficence of God.* By GEORGE FOWNES, PH.D., Chemical Lecturer in the Middlesex-Hospital Medical School. New York: Wiley & Putnam. Philadelphia: J. W. Moore. 12 mo. pp. 158. 1844.

Of the many definitions which have been given of man, the most philosophical is, that he is a *religious animal.* While the races below him, so far as we can judge, are limited in their wishes to the "ignorant present time," happy if their physical wants are supplied, and without the sustaining influences of hope in times of distress, man in all circumstances looks to the future. How happy soever may be his lot at any given moment, if his future be darkened he is made miserable. This involves the necessity of a belief in a future state where the deficiencies of the present life will be supplied, and the evils incident thereto will no longer exist. Such a belief, we find, has obtained in all ages, and among all nations, and may therefore be termed instinctive. Far from having always been wisely directed, it has in a vast majority of instances degenerated into a dark and cruel superstition; but it is nevertheless the paramount belief of the human understanding. "The first and most indispensable condition of happiness to a reflecting, intelligent man," remarks the author of the admirable little work the title of which heads this article, "must be the acquisition of sound, consistent, and cheerful views of the relation in which he stands to his Creator; without these, all external appliances and means are worthless. Unless he be deeply impressed, thoroughly imbued, with the conviction that he is, and shall be, every moment of his existence in the hands of a Being of unmixed and unbounded benevolence, exerted under the guidance of wisdom and knowledge, embracing all possible contingencies, and rendered effective by power extending to all things not involving contradiction, he cannot be permanently happy." Such a conviction the study of nature is calculated to impress upon the mind, and hence good men for centuries past have inculcated the study of the works of creation as affording the most convincing proof of the wisdom, power, and beneficence of the Creator. The writings of Boyle, who was equally conspicuous for his learning and piety, contain many arguments on this point. An admirable work has come down to us from the pen of John Ray, entitled the "*Wisdom of God manifested in the Works of the Creation;*" in which he considers the heavenly bodies, the elements, meteors, fossils, and vegetables, as well as the earth, its figure, motion and consistency, and especially the admirable structure of the bodies of man and other animals, in reference to that question. The same line of argument has been happily enforced by the pen of William Derham, a divine of a philosophical turn of mind, whose writings are still extant. The argument was taken up by the celebrated Dr. Paley, whose treatise on natural theology has acquired something of a professional character from the illustrations of Paxton, the anatomist. Since the appearance of that masterly work, the subject has received a flood of light from some of the first minds of the age. The hand has been exhibited by Sir Charles Bell as evincing design; Roget has treated of physiology; Kirby of the history, habits, and instincts of animals; Prout of chemistry, meteorology, and the function of digestion; Buckland of geology; and Whewell of astronomy and general physics, in the same relation; and Kidd and Chalmers have completed the argument by showing the adaptation of external nature to the physical, moral, and intellectual condition of man. The volume before us may be regarded in the light of a sequel to this series of works, known as the Bridgewater treatises. Chemistry is a most progressive science, and the new revelations which it makes from year to year of contrivance and design, form an interesting accession to the immense pile of facts with which the argument has been fortified. We propose taking up the subject somewhat in detail, and, for the sake of variety, occupying a few pages of our Journal with other matter than that which is strictly medical; and in doing so we hope we shall have the indulgence of our readers, who will agree with us in applauding the sentiment of the Roman dramatist, that nothing is beneath our regard which relates to humanity.

One of the most abundant, as well as one of the most interesting substances of which chemistry treats, is atmospherical air, which is indispensable to the existence of all organized beings. The tree soon dies if its roots be surrounded by stagnant water and the oxygen of the air fail to mingle with its circulating fluids; and the zöophyte fastened to the rock is as dependent upon the vivifying principle of the atmosphere as the most perfect class of animals. The first act of the new-born babe is to inhale the air, and the last effort of the expiring man is to command its receding wave. This fluid surrounding the earth to the distance of about fifty miles, the home of everything that lives, the laboratory in which all the multiplied and curious processes of nature go on, has been studied by men in all ages, but until modern times studied to but little advantage. It was long regarded as an elementary body, which united with earth, fire and water, to compose the endless varieties of matter. Only a little more than half a century ago it was not known what the principle was to which it owed its life-sustaining qualities. Dr. Hooke, in the time of Sir Isaac Newton, conjectured that it must contain something analogous to the principle in saltpetre which rendered that substance so effective in supporting combustion; but oxygen, the common principle of the atmosphere and nitre, to which they owe the power of maintaining flame, was discovered by Priestley late in the eighteenth century. The other element, nitrogen, had been discovered by Rutherford two years before Priestley separated oxygen, but he did not make the discovery that it was one of the constituents of the atmosphere. This was reserved for the great French chemist, Lavoisier, who, if his life had been prolonged, would probably have added to this other discoveries which would have left less for the genius of Sir Humphrey Davy to achieve. These more palpable truths connected with our atmosphere having been revealed at a period comparatively so recent—as compared with the age of physical science only but as yesterday—it is not surprising that there are still phenomena pertaining to the air which have eluded the scrutiny of the whole body of chemists, up to the present hour, and which fresh researches and more accurate analyses are necessary to unfold. The vapor so extensively felt, so much dreaded, under the influence of which so many thousands of our race are now racked with fevers or shaking with agues, how has it escaped the search of the philosopher! *Causa latet; vis est notissima.* The chemist has sought for it in vain in the most pestilential airs—over marshes and rice-fields, where to breathe the atmosphere for a single night is to contract a malignant fever. Art has not yet furnished us with the means of recognising any ne gas even in places where the fever poison is so palpably present.

But enough is known of our atmosphere to prove that in its constitution and arrangement it is pefectly adapted to the wants of the beings that respire it, maintaining a comparative purity in the midst of circumstances which tend perpetually to render it impure; continually giving up one of its elements in large measure, and yet retaining each one in a state of perfect equilibrium. Not only is it indispensable to the beginning and perpetuation of life upon the earth, but it is the only fluid which could support the respiration of animals. No other combination of gases, though some of them would be innocuous, could replace the present elements which compose the atmosphere. The vitalizing constituent is its oxygen, to breathe which all animals are provided with lungs or gills, or some apparatus by which it may be made to touch the blood. It gives vitality to the circulating fluid, suffuses the human cheek with the ruddy glow of health, and imparts elasticity and vigor to the muscular system. Nitrogen, the other constituent, is an inactive element, taking directly no part in the animal functions, entering into no combination with its fluids, and serving no other purpose that can be perceived but to dilute the oxygen, which would be too exciting to the system if uncombined. But, then, nitrogen is the only diluent, out of many gases, that could have been employed in an atmosphere designed for the living race. Hydrogen was substituted by Pilatre de Rozier for a little while, but his artificial atmosphere was combustible, and when he set fire to it an explosion resulted by which he thought, at first, that he had lost his teeth. In an atmosphere of hydrogen and oxygen, animals become drowsy as when exposed to the narcotic gases. In such an atmosphere, even if he did not perish speedily, man would lose his energy and grow listless and stupid, and a universal sleep would fall upon the animals that now sport around him so full of life and vigor. But not only so; as these gases form a fulminating compound they would take fire the moment that an ignited body was brought into contact with them, and explosions fatal to all animal existence would ensue. Fires would be out of the question in an atmosphere so constituted, and if a spark should chance to fall into it, or the clouds transmit their lightning through it, a conflagration would be kindled up which would shake the globe to its centre, and in an instant precipitate the air to the earth in the form of water. It is therefore clear that hydrogen could not take the place of the passive azote, the very passiveness of which seems indispensable to it as an element in our atmosphere. And it may be added, that of the many elementary bodies known in chemistry, all of which are capable of taking on for a time an aerial shape, no two but oxygen and azote would form a respirable fluid answering to all the exigencies of the living world. Most wisely, we conclude, then, has it been composed. Not the best materials merely, but the only materials extant in our world that would answer, were employed in its fabrication.

Nor is this all. The proportions in which the elements have been combined are precisely the proportions requisite to the maintenance of life in full vigor and for its present term. If oxygen were more abundant in the air than it is, the systems of animals would be unduly excited; fever would ensue; and, as the flame burning with unwonted intensity sooner exhausts the matters it feeds on, the living body would perish of rapid inflammation. The life of man, limited now to a few score years, would be narrowed down to a few days of delirious existence. Even if he might be inured to so exciting an atmosphere, and escape the consequences of a combustion in his own frame, he would not be able to regulate his fires, or to employ many of the matters which are indispensable to his comfort. All his utensils of iron would take fire and become useless when he attempted to mould them by heat into the desired form. The various combustibles that now burn with a quiet and steady flame, diffusing light and cheerfulness through his dwellings, would pass rapidly away in an intense glare of heat and light unsuited to his purposes.

But this augmentation in the amount of oxygen is not the only disturbance that might be created. We may suppose the oxygen wasted and the nitrogen predominant, in which case we should have an air laden with a soporific influence—an air in which men might live, but their lives would be such as cold-blooded animals live. The system would languish under the want of its necessary stimulus; the blood, no longer crimsoned by its wonted contact with vital air, would creep in a purple current along lifeless tubes; the brain, robbed of its needed stimulus, oxygenated blood, would grow feeble and dull, and mind would no longer put forth its accustomed strength; the wings of the imagination would become powerless and tame, poetry would lose its inspiration, eloquence would cease, and even judgment, slow now in its operations, moving with a cautious and wary step, would lose all power of action. So that whether we should remove either of its constituents, or simply vary their proportions, whether we should exalt or reduce its tone, disorder would be equally the result; and since this peculiar constitution is in perfect harmony with the sentient beings of this globe, the inference is natural that it came from the hand of a benificent Creator.

This evidence of wisdom and design becomes stronger when we look more closely at the arrangement by which the air is preserved against deterioration. The balance in its elements, so exquisite that the chemist has sought in vain for any variation, is opposed by multitudes of agencies which tend continually to disturb it. Every fire that burns, and every animal, during every moment of its existence, wastes one of the constituents of the air; and but for a provision counteracting this waste, the healthful atmosphere in which we now find ourselves invested would long ago have given place to an assemblage of vapors more pestilent than the air of an infected city. What is this provision? How, amid all the disturbing forces which ceaselessly assail this balance—the countless fires, and the myriads of animals that imbibe the oxygen, while they load the air with another gas hostile to life—is the primitive condition of this fluid maintained;—the oxygen kept from declining, and the carbonic acid, evolved without intermission over all inhabited lands, restrained within limits that render it harmless? By what magic influence is this beautiful adjustment preserved in the midst of this perpetual strife, so that after a contest of six thousand years neither force has gained any ascendancy?

At first the contest appears to be a perilous one, for as the carbonic acid is much heavier than atmospherical air it should tend continually to displace it, and by lifting it above the earth remove it from beyond the reach of men and animals. But what, looking at it *a priori*, would seem to be a dangerous tendency, is obviated by another law, which controls the law of gravitation. As late as the time of Lavoisier this law was not understood, and that acute philosopher believed that the light gases floated above the heavier, such as were combustible being kindled, now and then, in the upper regions of the atmosphere, and giving rise to the coruscations of the aurora borealis. According to this law, the full development of which we owe to chemists of our own times, each gas is independent of every other, and expands and diffuses itself as freely as if it alone existed. The particles of carbonic acid rise through the oxygen and azote as freely as if it were rarer, instead of being, as it is, much more dense than those gases are. Each seeks an equable diffusion, and uniformity of constitution in the atmosphere is the result. No one constituent can ever accumulate at one point, or be permanently diminished at another. The particles of oxygen resting upon other particles of the same fluid are quickly sensible to the change, when its volume is affected by any of the disturbing causes to which we have alluded, and a motion takes place among them by which the vacuum is filled. In crowded cities, where numberless fires and lungs are rapidly abstracting oxygen, there is a tendency to a loss of the equilibrium—to a deficiency of the vital element and an excess of the azote, as well as of carbonic acid, which the lungs and fires evolve in copious measure. But the loss is never perceived; the noxious redundancy rarely occurs; it is only in peculiar situations that carbonic acid accumulates to a dangerous extent. Oxygen from other regions, from over the green fields of the country, and the distant mountains and seas, pours in perpetually to supply the waste; while the carbonic acid spreads abroad and attains a general diffusion. And thus it comes that the city has an atmosphere as free from these noxious gases as the air of the country, or only so slightly tainted by their presence that the chemist detects no increase in the proportion of its carbonic acid, over what is found in air taken from the summits of lofty mountains, or that which has been brought down by the aeronaut from its most elevated fields.

But there is still another provision for maintaining the equilibrium between the elements of the atmosphere and this intrusive gas; a provision as evincive as anything that could be conceived of beneficent design. This is the relation of the vegetable kingdom to carbonic acid, which is such as to form a perfect balance between the two departments of organic nature. The functions of animals involve the production of this gas, and vegetables cannot grow without it. It is the appropriate and indispensable food of plants; they inhale it when in a growing state perpetually from the atmosphere, and, fixing the carbon in their tissues, set the oxygen free to minister again to the wants of animals. In this manner the atmosphere becomes the source whence vegetables derive their chief aliment, while these, by absorbing and decomposing the carbonic acid, contribute to the purity of the air, the forests and the fields returning to it its vitalizing element. By all these contrivances the balance is maintained with undeviating exactitude; nor, while these laws continue to be operative, can any derangement occur. No deleterious vapor can remain long stationary in any place owing to the law of diffusion, and so the poison of the pestilence becomes diluted and loses its power. It is impossible for the air ever to be stagnant. Its constituents, as we have seen, penetrate each other readily, and are continually changing place; its particles cohere by a trifling force, and hence currents are ceaselessly formed. That portion which grows heated is rarified, and rises, while the colder and heavier columns from above descend to take its place. The volume which rests at one moment near the earth, the next, expanded by the heat of the sun, may be ascending towards the region of frost, to return again to the earth after it has grown heavier by growing colder, and breathe among the flowers in the warm valleys below. The wave which floated yesterday over an iceberg at the poles, may be moving to-morrow over the sunny plains of the south; while the sultry air of the equator is passing in opposite and more elevated currents to temper the inclemency of northern skies. Thus the aerial ocean is never at rest. Currents are set up at every moment of time, and are a part of the machinery by which the air is preserved untainted.

Another quality in the gases composing the atmosphere displaying equal wisdom and design, is their low power of transmitting caloric. Among the conductors of heat the gases are the worst; and it is even questionable whether they can be said in any true sense to conduct it at all. This negative property is essential to the air as the habitation of beings possessing a fixed temperature. Warm-blooded animals perish if the heat of their bodies be increased only a few degrees, and in a medium conducting heat rapidly such a result would be continually threatened. And in like manner the extinction of the living races would be endangered by the rapid abstraction of their vital heat in cold weather. Water heated above 120° of Fahrenheit's thermometer becomes painful to the hand; but men have exposed themselves to the air of rooms heated more than twice as high. Iced water would soon produce a fatal reduction of the temperature of animals; and yet men have gone abroad without injury when the air was a hundred degrees colder; and tribes of men subsist in climates where the winters are sufficiently intense to congeal mercury. If the atmosphere had been endowed with greater powers of imparting and abstracting heat than it possesses, it is manifest that it would not have been fitted to sustain the animal races, that now preserve their own unvarying temperature amid all the vicissitudes to which it is annually exposed. Just effective enough by the interchange of its particles in mitigating the heat of the tropics, it is so slow in robbing the body of its caloric that men find it practicable to live in the most rigorous climates.

Water is not less affluent than the atmosphere in facts evincive of beneficent design. Water, indeed, is most curiously made, and the more we look into its constitution the more we find in it to admire. It consists of bases which kindle the most intense flame at the moment of their union, and, the antagonist of fire, is formed whenever the fire burns. Its bases when they unite with other elements often give rise to compounds possessing acid properties, and no acid exists in which oxygen or hydrogen is not found to be a constituent. But when these elements unite with one another, instead of an acid, we have a bland fluid, destitute of taste and smell. The air, we have been seeing, is in admirable harmony with the sentient beings that breathe it; but it would be a most unfit agent of respiration but for the presence of the water which infiltrates it like another atmosphere. Deprive the air of this aqueous vapor, and what is now the evening wind, or the refreshing sea-breeze, would become the blasting sirocco. But evaporation is so active that parched winds can traverse only narrow tracts of the globe. Wherever water exists evaporation goes on, and is not suspended even when the water becomes ice. In winter and in summer, in the burning and in the frigid zones, from the humid earth, and from expanded seas, the silent, invisible vapor is continually ascending. It is computed that the Mediterranean in this way gives up more water than it receives from all its great rivers. This water might have floated continually in the atmosphere as a cloud or fog, in which case, although it would have served as a medium of respiration, it would have drawn a perpetual veil across the heavens, shutting out from us the glorious light of the sun and stars. And so it has been kindly arranged that after the storm sunshine shall succeed. The vapor which rises spontaneously from water is not suddenly condensed like that which is raised by artificial heat, but in ascending combines with electricity, whereby its particles are rendered self-repellent, and assume the form of minute vesicles. These little hollow spheres constitute clouds, which are buoyant by virtue of the electricity with which they are charged. So long as the vesicles preserve their form the water of the clouds is kept suspended, but the moment they collapse the rains begin; and this takes place when the charge of electricity is lost. The earth being in an electrical state opposite to that of the clouds, will attract them; their electrical fires will pass to the earth; and thus the storm begins. The showers always descend most copiously just after a flash of lightning, the cloud-bubbles collapsing in consequence of the escape of the electrical fluid. But the balance is restored after a time; the lightning is no longer in motion; spots of azure break out in the troubled sky, and the "bow of hope and promise" starts up upon the bosom of the retiring cloud. Mountains favor this process, and in view of their beneficent agency might be reckoned to be so many guardian giants, which have been stationed through the earth to collect the showers and disperse them over the valleys and plains consigned to their care. As more rain falls in mountainous districts than in champain countries, they give origin to numberless rivulets, which gradually swell into broad rivers that bear upon their bosoms the commerce of the country, and carry fruitfulness to the lands below.

But the whole beauty of this machinery has not yet been unfolded. Water, it is well known, comprises much the larger portion of the surface of our globe, and these vast oceans are sources of perpetual exhalation. What would be the result, if the water thus exhaled into the air constituting the material of clouds were to return directly to the bosom of the deep? It is quite palpable that barrenness would spread over the earth, and all organized beings perish from the face of it; and the arrangement by which this is prevented must be held to evince benevolent design. The clouds of the ocean, probably because charged with the same kind of electricity as the ocean itself, are repelled from it; the vesicular form of the vapor is preserved, and the buoyant clouds float over the sea to the distant shores; there to be drawn down by the hills and mountains, watering the earth that it may give bread to its inhabitants; again to be borne by the universal slope to the parent ocean; and so continue to run through this magnificent circle of changes so long as the existing condition of the world endures.

Let us consider for a moment some other interesting phenomena connected with the precipitation of atmospherical moisture. All are familiar with the fact that bodies, suddenly cooled, condense the aqueous vapor in the surrounding atmosphere, and most persons have been struck with the connexion between the ready condensation of this moisture on the tumbler of cold water, and that state of the weather in which rain is likely to fall. The earth being a better radiator of caloric than the air, is found soon after sunset to be colder than the air just above it; and thus it becomes a condensing body. The vapor in the air surrounding it is precipitated in the form of dew. But who has not been struck with that beautiful provision made for the living world, as he has walked abroad in the morning and beheld each spire of grass, and every green leaf pendant and dripping with the dews of the night, while the dry inorganic bodies around had received only a small share of the moisture. The plants, owing to their rough surfaces, give up their caloric more rapidly than the dry, hard earth, and for that reason the dew is distilled more richly upon them.

Although water evaporates at a very low temperature, the evaporation is in a direct ratio to the heat; and hence it follows that in hot climates it is much more rapid than in cold. It is most abundant in tropical countries, where it answers the beautiful end of mitigating the intense fervor of the sun; and is but trifling in amount in polar latitudes which have no heat to spare. And the mode in which it descends differs equally in the two regions. In northern climates it comes down in a gentle, almost perpetual mist, and warms the air by the transition from a vaporous to a liquid state. If it fell thus in hot climates, the clouds continually hovering over the earth would check evaporation, and render the air intolerably sultry; the rains falling so slowly would sink deep into the ground, and vegetables bathed in this perpetual moisture would rot before they ripened; and the face of the country would become an extended marsh, exhaling every fetid and poisonous effluvium; a fit residence only for lizards, bitterns, and other unsightly aquatic animals. But in such latitudes it descends in sudden, drenching rains which flow rapidly into the streams; the clouds quickly disburdened of their showers are soon dispersed, and the vertical sun beaming upon the earth renews the process of evaporation, dissipates the excessive moisture, and enlivens the inhabitants with an elastic atmosphere.

The specific gravity of water is precisely that which fits it for the various offices it must perform in the present constitution of our universe. By virtue of its density it floats the seeds of vegetables to plant new colonies upon distant continents and islands, extending thus the dominions of the living world. The ocean, too, is whitened with the sails of commerce; the light of science, and the arts that soften and refine, are borne to every quarter of the globe; and civilization, and the hopes of the gospel are spread as far as there are winds to waft or seas to roll them. A fruitful soil is brought down from the disintegrating rocks of the mountains, and spread out into vallies and plains for the use of the husbandman. A home, adapted to their forms, answering to all their wants, is furnished for the myriads of aquatic animals which swim upon its surface or glide swiftly through its waves. Increase its density, as to that of quicksilver, for example, and ships of iron with platinum for ballast alone could navigate it in safety. Or make it lighter, and the light canoe would not float upon it, and the splendid barque that now "walks the water like a thing of life," would be engulphed and lost beneath its waves.

One of the most admirable laws of water is, that it freezes. This subserves a hundred most important purposes. Water gives out heat in freezing, and thus in the coldest quarters of the globe, as it is congealed by frost, it is liberating vast volumes of caloric, and the process of congelation is in this way counteracted, and at last arrested, by the heat which itself sets free. Freezing is a warming process. Every fall of snow, it has been computed, imparts more heat to the atmosphere than would be afforded by the same quantity of pulverized red hot glass. But evaporation is a cooling process. Extended oceans spread their bosoms to the sun, in equatorial regions, and the vapor which they send up tempers the fervor of his vertical rays. It is borne along to the north by the upper currents in the air, and descending in the form of sleet or snow, sets at liberty the heat which it absorbed in the south. Thus by the same splendid operations which cool the temples of the children of the sun, are the horrors of the polar winter softened and made supportable. The aurora borealis, the glory of northern climates, is also an effect of the same arrangement. The electricity which is transferred thither in combination with the vapor, descending with the caloric and accumulating upon the ice at the poles, at last breaks through the atmosphere into the vacuum above, and in hastening back towards the tropics gives out light to cheer the long winter nights of those regions.

Other advantages grow out of the congelation of water, and the circumstances which attend this process. Most bodies grow smaller as long as they continue to grow colder, contracting when they freeze. Water is of a smaller class which obey another law; a law fruitful in most beneficent consequences. It expands in freezing; by which we see bottles holding it broken; strong tubes conducting it burst; and the strongest metals confining it riven asunder. Mark, then, the operation of this law. The earth, after receiving the rains of autumn, is congealed by the winter's frost. The water in it being expanded, the soil is pulverized and made light, and prepared for the roots of the tender, growing vegetables in spring. But more. Ice, as a consequence of it, forms first on the surface of our rivers and streams. The intestine motion which goes on in water when subjected to a change of temperature ceases at a particular point. Cooled down to 40° of Fah., it attains its maximum density; and as the cooling proceeds, those particles which are reduced below this point float upon the surface, being lighter, and finally arriving at 32° are frozen. In the mean time the warmer water below being heavier, remains at rest; its heat being in some degree kept in by the covering of ice above. Did water continue to grow heavier, as it became colder, until it arrived at the freezing point, a cake of ice would form first at the bottoms of our rivers; the whole of their waters would be reduced to the point of freezing before congelation began; and their fish by affording a nucleus would be among the first objects to be incarcerated by the hardening ice. The cold of a few nights would congeal deep rivers, and the ice lying far below, would not be softened by the returning suns of summer. The inhabitants of water would perish. Coasts would cease to afford, as now, the most genial climates and luxurious homes.

So much turns upon the simple law, that water grows lighter before attaining the point of congelation, and expands in freezing.

Water freezes at the right temperature. Alcohol has never been frozen by the cold of the polar winter, or by the most intense freezing mixture. Suppose such were the law of water. We should indeed have frozen oceans no longer, and the explorer of northern seas would find a liquid surface as far as there were gales to propel his ships. But we should have rains much colder than ice or snow,—rains that would sink the thermometer to zero—that falling upon the earth and penetrating the soil, would chill it beyond what the heat of the returning summer could restore, and blight and destroy the seeds and roots of all vegetation.

But now, reduced to the temperature of 32°, it loses its liquid form, gives its latent heat to the ambient atmosphere, and descending as fleecy snow, throws around the earth a mantle which shields it from the more intense degrees of cold. For snow is a very imperfect conductor of caloric. The earth is often little lower than 32°,—of the temperature merely of ice—while the air is 50° below zero.

But inconceivable disasters would flow from its easier congelation. The cold of a night might arrest commerce—the wide ocean would be covered with ice, our springs cease to run in winter, and a solid crust bind up the earth where now the plough of the husbandman finds a pulverized and genial soil ready to receive the seeds of the future harvest.

No common liquid undergoes ebullition at the same temperature with water. Ether boils at 98°, alcohol at 175°, mercury at 650°, water at 212°. If it boiled at 98° with ether, we should be frozen in mid-summer, as we freeze water by the evaporation, in other words, by the boiling, of ether under the exhausted receiver of the air pump. If it boiled, with alcohol, at 175°, still the earth and our bodies would be too much cooled by the rapid evaporation; while we should be forced every day to complain, with the Augustine Monks on the great St. Bernard, that the water could not be made hot enough to prepare our soup. But if, instead of boiling at these reduced temperatures, it reached the point at which mercury boils, before ebullition commenced, it would disorganize and destroy all materials of aliment, and become useless as a culinary agent.

But, although the boiling point of water is so much below that of mercury, its capacity for heat is about thirty times as great. Thirty times as much heat is necessary to raise water to 212° as to bring mercury up to that point, and consequently water is heated thirty times more slowly than that liquid. In boiling, water combines with much heat which becomes latent, and is not appreciated by the thermometer. The same is true of ice in melting. Hence the boiling of water, and the melting of ice, are processes which go on slowly. Were the law otherwise, and did not the great capacity of water for caloric exist, when it became heated up to 212°, it would instantly flash into steam, and no boiler, however powerful, would be able to resist an explosion. And in like manner ice would instantly liquify, when it was raised to the temperature of 32°. Instead, as now, of softening gradually under the increasing heat of spring, the accumulated snows of winter would be melted by the heat of a single day, and pour down from hills and mountains in wide-wasting inundations.

Water is the natural beverage of man and other animals, and as a diluent for their food it is necessary that it should be comparatively free from saline substances; and yet the sea is charged with salts. The chloride of sodium in which it abounds, like water, is a necessary of life; as a condiment it is essential to the tone of the alimentary canal, the privation of which induces disease. Besides, it is an antiseptic upon which we depend mainly for the preservation of much of our most valuable food. The ingenious author of the work referred to at the head of this article conjectures, that the water of the ocean was not originally brackish, but has acquired that quality from the washing of the land by the rivers which flow into it. However this may be, we find the condition of the sea, at present, precisely such as favors the wants and business of human society. The salt in its waters tends to preserve them pure, while it is an abundant store-house from which men may draw forever; it renders the water heavier, thus adapting it more fully to the purposes of commerce; it also makes the water much more slow in congealing. This ingredient, so important where it is, is left in its ocean-bed while the water is exhaled. Fixed in its character, heat never evaporates it, and the vapors which rise from the sea are without any saline admixture.

The earth is equally full of these proofs of design. We speak not of its position in reference to the sun, nor of its mountains, and slopes, and valleys, which give us the variety of seasons and climates, and our oceans, and running streams; but of its chemical constitution. Entering into its countless forms of matter, there are recognized nearly sixty elementary substances; some of which, in their action upon organised beings, may be said to be neutral; some are capable of assuming shapes which minister to their nourishment and growth; some exist in quantities so small that they are rarely detected; many can be isolated only by delicate and tedious processes; and many, in respect to animals and vegetables, are highly noxious. Silica is the most abundant, after oxygen; and, to say nothing of the necessity of this substance in the composition of the cerealia and many other species of plants, whereby their stems are rendered hard and capable of supporting their ripening fruits, or of its importance in many of the most useful of the arts, such as glass making, porcelain, bricks, &c., it is the only material known in chemistry out of which the soil could have been formed. It renders the soil porous and penetrable by the rootlets of plants. It allows the rains to descend into the earth to water the growing tribes of vegetables. The matter associated with it in largest measure is alumina,—familiarly known as clay; and these two constitute a bed in which the water holding the food of plants is detained sufficiently long without being kept about their roots permanently, as it would be in a bed of pure alumina. They form a perfect soil, so far as relates to the retention of moisture; but the clay and sand are but little concerned in the development of plants, and although essential ingredients in the soil, and forming much the larger part of it, if other elements had not been associated with them the earth would have remained a barren waste. Lime, in the form of a phosphate and a carbonate, enters into the composition of vegetables and animals, and to the latter is an indispensable article, since of it is chiefly composed their bony frame-work. Without phosphoric acid and lime, we are safe in saying there could not be any vegetable or animal life, and accordingly we find that these substances make a part, though often only a very inappreciable part, of every rocky bed. Potassa is equally indispensable to the maintenance and development of organized beings. Every part of plants contains it, and it is also a constituent of many animal secretions. So obscure was the origin of potassa and phosphoric acid, that for a long time physiologists were inclined to maintain that they might be created in the living organism by the powers of life; but at the present day this notion is entirely discarded, and no one now doubts that they are derived by plants from the soil. The crust of our globe, after we descend below the sedimentary deposits of limestone, shale, marble, &c., is formed of granite, a large proportion of which consists of felspar, and of this 14 or 15 per cent. is potassa. The source of this alkali is therefore perfectly evident. Chemists had more difficulty in detecting phosphoric acid, but Fownes has at length succeeded in showing that it exists in clays, and is a constituent of granite. Felspar, though an exceedingly compact and resisting rock, gradually crumbles down under the influence of the weather, by which the contained alkali is rendered soluble. The disintegration goes on so slowly that no more potassa is set free, from year to year, than is needful for the support of the annual growth of vegetables; the wisdom of which provision will be seen when it is considered, that the alkali is easily soluble and would be wasted by the rains if disengaged more rapidly than plants were able to take it up; in consequence of which the land would become barren. The potassa thus imbibed with their water by vegetables is easily extracted from their ashes, and forms an article of the highest economical value. The price of soap and of glass, not to specify other products of human industry, would be enhanced many fold if the supply of potassa were cut off, as virtually it would be were we compelled to extract it from clays, instead of deriving it from the ashes of vegetables. It is the presence of this alkali in milk that keeps it fluid; when neutralized by an acid the milk coagulates; and since fluidity is a necessary condition of the food of young animals, it is clear that but for the existence of potassa in the earth, from which plants extract it, the preservation of the animal species upon the earth would be impossible.

Of the fifty-five or fifty-six elements known to the chemist, more than forty are metals, and while many are objects of great interest and value, not a few are possessed of such qualities that their wide diffusion through the earth would be a dire calamity. Carbonate of lime is met with as forming a portion of the rocky strata of most countries; the water used by the inhabitants of such countries running over these strata is highly impregnated with this inactive salt. Now suppose, in place of these limestone beds which cover millions of miles of our earth's surface, the external crust consisted of carbonate of baryta? Manifestly, the "pure antediluvian beverage" would have been rendered unwholesome by the presence of this compound, and the race of animals would probably, long since, have perished from the earth. In like manner, if arsenic had been extensively mingled with our soils or blended with the superficial strata of our globe, the result to animals would have been disastrous. But there is one metal so inoffensive in its character that it might safely enter into every soil. Belonging to this beautiful class of bodies is one without which human society could never have become refined or powerful; without which art could not have attained its efficiency, nor science its marvelous elevation; without which fields must have been unskilfully tilled, and navigation limited to the humblest coasting excursions. It need hardly be said that we refer to iron, for our description is applicable to no metal but that:—a metal, which is not only the most valuable of the class, and incomparably so, but is at the same time much the cheapest—diffused more widely than our own restless race—piled up in mountain masses in many countries;---a metal which takes precedence of all others in many mechanical processes, because it is the hardest, the toughest, the most enduring. Iron is, indeed, a wonderful metal; naturally the plainest and the least showy, but susceptible of a lustre outshining all its kindred; the only metal which, by collision with the flint, emits fire; almost the only one which bears welding; the only native magnet, and nearly the only one in which magnetism permanently abides—forming the needle by which the mariner, in the darkness of the night and amid the fury of the storm, guides his barque in triumph to the distant haven; the only individual of this extended class which, in all its forms and combinations, is friendly as well as safe to the economy of animals, making one of the essential constituents of their blood, and for their health gushing in a thousand springs. It is curious that iron, which has been applied to so many purposes of usefulness and luxury, should have been found to be an efficacious antidote, and, in the present state of our knowledge, the only antidote, to arsenic; and still more curious, that it should turn out to possess the power of disarming even a more speedy poison of its virulence. As if not enough that iron should be "king of the prow, the ploughshare, and the sword," and the antidote to arsenic, it is endowed with the ability to divest prussic acid of its noxious qualities.

The human race was slow to discover the virtues of iron, and ages passed away before it was generally applied to the wants and purposes of civilization; but when it came to be searched for and extensively used men were surprised to find how their convenience had been consulted in its distribution. Deposited in inexhaustible beds in the sides of mountains hard by the iron, are almost always found extensive coal fields; the metallic ore and the fuel being placed in alternate or contiguous strata. And often in the same neighborhood the limestone rock is found which is necessary in the extraction of the metal. This arrangement is so convenient, so wise, that it cannot be regarded as accidental; but we must conclude, that the hand which deposited the iron ore in the hills, laid down there also the strata of stone coal and of carbonate of lime, to be used by man, in remote ages, for separating the valuable metal from its dross.

The origin of these beds of coal is a problem which has greatly perplexed geologists; but whether we suppose with Adolphe Brongniart that they are the remains of plants which were necessary in the early stages of our globe to extract from the atmosphere the excess of carbonic acid, thus fitting it to become the theatre of the higher races of animals; or conclude that they consist of the combustible parts of plants which grew and flourished in an atmosphere having the constitution of the present one, no one can doubt that they are of vegetable origin; and as little can any one fail to see in these extended deposits of fuel a regard to the comfort of those who were to become the rational inheritors and lords of the earth. We say, no one can question that the coal fields, immense as they are in superficial extent, and rising thousands of feet in height, are the residuum of what were once living vegetables. Whether or not we shall ever be able to make out a satisfactory solution of the manner in which they grew, and perished, and were submerged, one layer after another, until the mass became mountainous, we are forced to adopt the belief that what is now the amorphous stone coal had once the delicate organization of plants. These perished in their season, and their parts, protected against the oxygen of the atmosphere, have taken on this shape. There they have reposed for untold centuries, a mine of energy and wealth for the human race; and when they are extracted by the miner and submitted to the transforming force of fire, their elements are sent abroad once more to take a part in the business of life. Their carbon, uniting with the oxygen of the atmosphere, assumes an aerial form, and their hydrogen, combining with the same principle, ascends in the shape of invisible exhalation. This column of vapor rising from every fire is gradually condensed into clouds, and falls to the earth in fructifying showers; and the carbonic acid is also brought down again to hasten the growth of vegetables, to enter into their structures, expand in their green leaves, develope their fruits, and go abroad in the aroma of their flowers. The plants which adorned the earth when as yet there were probably no human eyes to enjoy their beauty, having slumbered for ages unknown, by this beautiful provision become once more active; and after warming us in the fire, and cheering our habitations by the light of their combustion, go back to the sisterhood of plants, there to pursue the round of growth, decay, and reproduction until it shall please Him who organized them in the beginning to summon them to their last repose.

It would not be easy to imagine any more conclusive evidence of design than is afforded by the perfect adaptation of the two organic races to each other, of which the arrangement just described is a beautiful example. By the law of diffusion the carbonic acid generated in the lungs, and which would remain in the cells there but for this law, presses out and mingles with the atmosphere. Its retention in the lungs would very soon become destructive of life; but its presence in the air is indispensable to the sustenance of vegetables. It forms not more than one part in every two thousand parts of the atmosphere, and yet the carbon in this, insignificant as it seems, amounts to more than that contained in all the existing forests, and all the beds of mineral coal in the earth united. In this gas is an inexhaustible store-house of aliment, from which vegetables may draw from age to age. During the winter season, when the forests are bare, and the fields no longer clothed in verdure, the carbonic acid, which is largely evolved by the respiration of animals, and the numberless forms of combustion in cold climates, is borne away towards the south by the prevailing winds, where a perennial vegetation stands ready to take up the excess. And, in this way, animals may be said to furnish a portion of the food to the plants by which, directly or indirectly, all they themselves are nourished. Nor is carbonic acid the only element of food which vegetables owe to the animal kingdom.

Among the products of vegetable life we find a class of bodies of exceeding interest to us, on account of their relation to animal nutrition. These are albumen, fibrin, and a third compound termed casein, which agree in containing azote. They are essential to animals, as composing the materials out of which blood is formed, and are justly styled the "elements of nutrition." Now, it is almost certain that plants do not derive this azote directly from the atmosphere, and the question arises, from what source then do they obtain it? Like the carbonic acid, the azote is supplied to them by animals. Animal substances in undergoing decomposition are remarkable for the generation of ammonia, a gas containing nitrogen as one of its constituents. We have only to conceive of the countless forms in which animal matter is perpetually exposed to this process over the wide earth, to have some idea of the extent of this supply. The ammonia thus exhaled without intermission into the atmosphere is dissolved by its hygrometric constituent, and brought down to the earth along with the rains. It percolates the soil, and is aspired by the plants, which decompose it and fix its nitrogen in their nutritious compounds. The contrast between the two living races is here most remarkable. The chemistry of one consists in building up organized compounds; that of the other in breaking these down. Vegetables imbibe ammonia and carbonic acid, and out of them fabricate the organic matter that animals must receive in order to subsist; animals appropriate these organic bodies to their uses, convert them "by a descending scale into compounds less and less complex, until at length they reach the inorganic condition, and once more become capable of assimilation by plants." And thus "a perpetual and unbroken chain of vital phenomena is established, the products of one order of beings becoming the sustenance of the other." Dumas has very happily contrasted these opposite functions of plants and animals in his "Balance of Organic Nature," thus:

| The Vegetable | The Animal |
|---|---|
| Produces the neutral azotized substances, | Consumes the neutral azotized substances, |
| ———fatty substances, | ———fatty substances, |
| ———sugar, starch, and gum, | ———sugar, starch, and gum, |
| Decomposes carbonic acid, | Produces carbonic acid, |
| ———water, | ———water, |
| ———ammoniacal salts, | ———ammoniacal salts, |
| Disengages oxygen, | Absorbs oxygen, |
| Absorbs heat and electricity, | Produces heat and electricity, |
| Is an apparatus of reduction, | Is an apparatus of oxidation, |
| Is stationary. | Is locomotive. |

The exercise of the animal functions implies a waste of the materials of the body, and demands a perpetual supply of aliment. From the moment that the young animal begins to breathe to the close of its life, the parts of its body are in a state of incessant change. The secretions remove what has become useless; oxygen is absorbed into the system at each inspiration and converts a portion of it into water and carbonic acid, which escape every time the lungs collapse; muscular exertion and the very exercise of the brain itself in thought, are attended by changes in matter, as necessarily as the evolution of electricity in the galvanic battery is accompanied by solution of the metal. The supply of this never-ceasing wear of the animal body is derived from the vegetable kingdom; but until the subject was taken up by Liebig, chemists and physiologists entertained vague notions as to what were strictly the nutritious principles in plants. We have already mentioned that these are now conceded to be albumen, fibrin, and casein; and these remarkable products of vegetable life are identical in composition. It is upon these that animals subsist, whether derived from animal or vegetable food. Nutrition is the same in the carnivorous and the herbivorous tribes. All alike are nourished by these azotized bodies. The herbivora must consume more food to find the requisite supply of these principles than the carnivora are obliged to do, because the nutritious principles are concentrated in the food of the latter; but the elements out of which the blood and all the organized parts of the body are formed are the same in both.

But when we come to analyze the food of herbivorous animals more minutely we find that fibrin, albumen, and casein are associated with other matters essentially necessary to their comfort and their very existence. We find that along with these are sugar, mucilage, starch and oil, some of which their economy imperatively demands. In the grains so much relished by animals, starch is a much more abundant principle than albumen; in the grasses, sugar is the most important element. The young of the mammalia depend in the first periods of their existence upon the milk of the mother; and besides casein, the blood-making principle, we find in this fluid sugar or oil, and, in the milk of the herbivora, both. These take no part in nutrition, but they have another office to perform as important as that of developing the frame of the young animal. This is the maintenance of animal heat.

The food of animals, therefore, is not all nutritious, nor is the only object of eating the supply of the wasting tissues. A certain temperature is essential to the perpetuation of animal life, and this is preserved by a combustion within the body of a portion of what is taken into it as food. Alimentary substances are hence divisible into two classes;—*the plastic elements of nutrition*, or the azotized principles; and *the elements of respiration*, embracing sugar, starch, gum, the oils, and some others. The first, as already mentioned, have the same composition, and no difference can be detected between the fibrin, albumen and casein of vegetables, and the same elements as extracted from the animal solids and fluids. Taken into the stomach, and absorbed by the lacteals, these principles are assimilated to the animal economy without any change in their chemical constitution. The fibrin and albumen of vegetables become the fibrin and albumen of the blood, and of the muscular tissue, and the brain of animals. The elements of respiration enter the system without taking part in its nourishment; they are followed by oxygen which passes through the lungs and combining with them converts their hydrogen and carbon into water and carbonic acid, animal heat being the result. If there be an excess of these they assume the form of fat, under the influence of a refined chemistry, and are deposited in the body as a store of fuel to be taken up and oxidized in seasons of scarcity, or when the increasing cold makes larger demands upon the calorific function. The nutritious elements also undergo this process of combustion, after they have served their end in the animal economy. The parts which have lost their vitality in the incessant round of changes are seized by the oxygen, resolved into carbonic acid, and in this way removed from the system where their presence would be injurious. And the effete tissues, which thus pass away to minister to the growth of vegetables, do not leave the animal system without further contributing to its comfort by liberating heat. As in our seas and rivers, oxygen is evermore engaged in removing impurities by a slow combustion; so in the animal economy it maintains this process without intermission, from the moment of birth to the end of our days.

But these organized elements of food are not the only materials, necessary to the development of animals, which we draw from the earth. Nobody now imagines that the female bird *creates* the phosphate and carbonate of lime, which compose the shells of her eggs. They are supplied by her food, and if this be wanting in them, the shells are soft. We have already adverted to the dependence of animals upon these salts of lime for the efficiency of their bony frame-work; and also to the importance of potassa to milk. Soda is found always combined with albumen, and is necessary to keep it in a fluid state. Iron is one of the constituents of the blood, and sustains such a relation to its health that we are safe in concluding that the tone of the system could not be maintained in the absence of this metal. Sulphur and phosphorus are met with in the protein compounds, and take part in the animal organism. All these are drawn by plants from the soil. Potassa is extracted from the granite rocks, and soda from common salt, which is diffused everywhere; iron is separated from its ores, and sulphur from its salts. The vegetable, by a process of vital chemistry which we cannot imitate, combines them with its albumen and starch, its casein and sugar, and moulds them into the form most pleasing to the animal appetite, most conducive to animal health and vigor. And then a Providence, evidently considering the welfare of the human race, has kindly placed between man and the vegetable kingdom his domestic animals who live to collect for him the materials of his food. The grass which nourishes his sheep and oxen contains all that is requisite for his nourishment; but he would die of starvation if driven to subsist upon grass. His cattle go abroad over his fields, gleaning from all wholesome herbs the principles which make his blood, and invigorate his frame. He thus has leisure for study and the cultivation of his nobler faculties.

It would be a waste of time to insist that in all these relations of the living races to the earth and to one another, there are indisputable evidences of a beneficent controlling Mind.

These evidences are everywhere to be seen, but, to adopt the closing thought of this admirable essay, nowhere throughout the whole creation is the goodness of the Almighty more conspicuous than in the means he has provided for revealing himself to his intelligent creatures, by conferring upon them these powers of discovering truth, and of appreciating its beauty and loveliness.

The "*Actonian Prize Essay*" is worthy of a place beside the very best of the Bridgwater Treatises. Its scientific details will interest every reader, and its noble moral reflections elevate and warm his heart.

